# Macroscopic and histopathological description of gastric lesions in horse, donkey, and mule fetuses in the last trimester of gestation

**DOI:** 10.1111/jvim.17193

**Published:** 2024-09-23

**Authors:** Angie Lorena Medina‐Bolívar, Rafael Resende Faleiros, José Ramon Martínez‐Aranzales

**Affiliations:** ^1^ Equine Medicine and Surgery Research Line (LIMCE), CENTAURO Research Group, School of Veterinary Medicine, Faculty of Agricultural Sciences Universidad de Antioquia Medellín Colombia; ^2^ Universidade Federal de Minas Gerais, Equinova Research Group Veterinary School Belo Horizonte Brazil

**Keywords:** equids, gastritis, intrauterine, prenatal, stomach

## Abstract

**Background:**

Limited information is available on gastric diseases in neonatal foals as compared with extensive studies in young, adult, and geriatric horses. Reports on fetuses are scarce.

**Objectives:**

Assess at necropsy stomachs of horse, donkey and mule fetuses in the third trimester of gestation to characterize lesions present during intrauterine life.

**Animals:**

Forty‐six fetal stomachs from both sexes (21 horses, 21 donkeys, and 4 mules) in the third trimester of gestation were collected from a processing plant immediately after slaughter.

**Methods:**

Measurements of longitudinal and transverse axes, weight and volume and gastric fluid pH were taken, and glandular and squamous mucosae were inspected. All findings of the gastric mucosa and measurements of the stomachs were presented descriptively. Groups were compared statistically, with significance level set at *P* < .05 for all evaluations.

**Results:**

All gastric contents had pH >5.8, and mules had larger stomachs and higher weights compared with horses (*P* < .05). Macroscopic lesions were classified as hyperemic, punctate, and erosive. Histopathologically, lesions were consistent with a chronic inflammatory process.

**Conclusions and Clinical Importance:**

Our study provides evidence of macroscopic and histopathological lesions in the gastric mucosae of equid fetuses in the last trimester of gestation. Relevant information for perinatology and neonatology is provided regarding the prevalence and classification of preulcerous lesions in equids before birth.

**Abbreviations**
EGGD: equine gastric glandular disease
EGUS: equine gastric ulcer syndrome
ESGD: equine squamous gastric disease
H&E: hematoxylin and eosin
MP: margo plicatus
PA: pyloric antrum

## 1 INTRODUCTION

The gastric mucosa in equids (horses, donkeys, and mules) is susceptible to injuries that can lead to various lesions, primarily in the squamous mucosa. In addition, susceptibility of the glandular mucosa to injury is attributed to the loss of protective mechanisms.^1^, ^2^ For this reason, gastric diseases currently considered distinct entities include equine squamous gastric disease (ESGD) and equine gastric glandular disease (EGGD), both encompassed within equine gastric ulcer syndrome (EGUS).^3^ These gastric diseases vary in prevalence because of their multifactorial nature^4^, ^5^, ^6^, ^7^ and have been extensively described worldwide, negatively impacting horse welfare and turn impacting the equine industry.^8^, ^9^, ^10^, ^11^ Most studies on gastric ulcers have been conducted in adult equids.^8^, ^12^, ^13^, ^14^ There are fewer reports of gastric lesions in foals, with prevalences of 22% in a necropsy study where lesions predominated in the squamous mucosa^15^ and 50% in an endoscopic study that identified ulcers and erosions in the same mucosa.^16^ However, most studies in this population have focused on foals > 48 hours of age,^17^ with the youngest reported case being a 24‐hour‐old neonate.^18^

Previous studies have not reported gastric ulcers in aborted fetuses or foals delivered through dystocic births, suggesting that these lesions are not common before birth.^18^, ^19^ However, several morphological changes have been described in the squamous mucosa during development process, including fetal and neonatal life.^19^ Gastric lesions in equid fetuses still are not clearly defined, so the associated risk factors remain unknown. Therefore, additional studies are needed to determine the prevalence of EGUS in neonates of various equid species.

Given the limited number of studies with small sample size and only including horses, we aimed to evaluate at necropsy stomachs of horse, donkey, and mule fetuses in the third trimester of gestation. These fetuses were obtained from pregnant equids subjected to stressful events before slaughter. Our objective was to document and characterize any lesions in the squamous and glandular gastric mucosae.

## 2 MATERIALS AND METHODS

Stomachs from equid fetuses were obtained from a processing plant immediately after the slaughter of the dams. All equid dams were considered to be creole breeds and originated from various regions of Colombia. The dams underwent long periods of travel and fasting and were exposed to stressful events (eg, mixtures of animals from different places, aggressive behavior, expressions of dominance among animals). Additionally, a necropsy study determined frequencies of 83.3% and 68.3% of ESGD and EGGD, respectively, in this population of dams (data not yet published).

Fetuses were selected based on age, including only those dams in the third trimester of gestation, which was determined by fetal size according to species, conformation and complete presence of a haircoat. With prior examination of the uterus, membranes and fetal fluids, they were selected and distributed by sex for each species.

After identification, each stomach was weighed, and measurements of the longitudinal and transverse axes, and the lengths of greater and lesser curvature were taken. A sample of gastric content was collected for pH measurement. Subsequently, a cut was made between the cardia and the pyloric antrum (PA) through the greater curvature to fully expose the gastric surfaces. Each empty stomach then was weighed, and inspection of the squamous or aglandular mucosa of the dorsal fundus, cardia area, and margo plicatus (MP) region was carried out. The glandular mucosa was evaluated, inspecting the regions of the ventral fundus, adjacent area of the MP and PA. The observed lesions were described based on their extension and appearance in both mucosae and classified as focal hyperemia with punctate lesions, multifocal erosion, or diffuse hyperemia.

After inspection and evaluation of the gastric surfaces, stomachs with lesions in the mucosae were selected by convenience sampling from 9 horses, 10 donkeys, and 2 mules. Samples were taken directly from the most obvious lesions found in each stomach. These selected stomachs underwent conventional histopathological analysis with hematoxylin and eosin (H&E) staining for description and comparison of the inflammatory processes (gastritis) and identification of lesion characteristics (erosion, hyperemia) among the species (horses, donkeys, and mules).

Macroscopic lesions were defined based on their characteristics as follows: reddish areas on each of the mucous membranes were identified and classified as focal or diffuse hyperemia according to their extent. Additionally, superficial loss of tissue continuity was identified and termed erosion. Microscopic lesions were classified as follows: hyperplasia (increase in the number of cells in the tissue compared with the adjacent borders), congestion (accumulation of erythrocytes within blood vessels), edema (diffuse accumulation of interstitial fluid), hemorrhage (extravasation of erythrocytes), lymphangiectasia (enlarged lymphatic vessels), presence of mononuclear cells, exocytosis (presence of material in vesicles) regardless of cell type, erosion (loss of tissue continuity), cystomatosis (dilatation of glands), and fibrosis (excess collagen).

All measurements were analyzed using descriptive statistics and are presented as means and percentages in comparative tables of frequency, severity, and macroscopic and microscopic description for each species. In addition, data were analyzed utilizing statistical software (GraphPad Prism 10), with significance level set at *P* < .05 for all evaluations. Normality was assessed using the Kolmogorov‐Smirnov and the Shapiro‐Wilk tests. Parametric variable values from each species were compared using an unpaired Student's *t*‐test. The frequencies of macroscopic and microscopic lesions across species were compared using Chi‐squared and Fisher tests, considering various types, grades, and anatomical sites.

## 3 RESULTS

**FIGURE 1:**
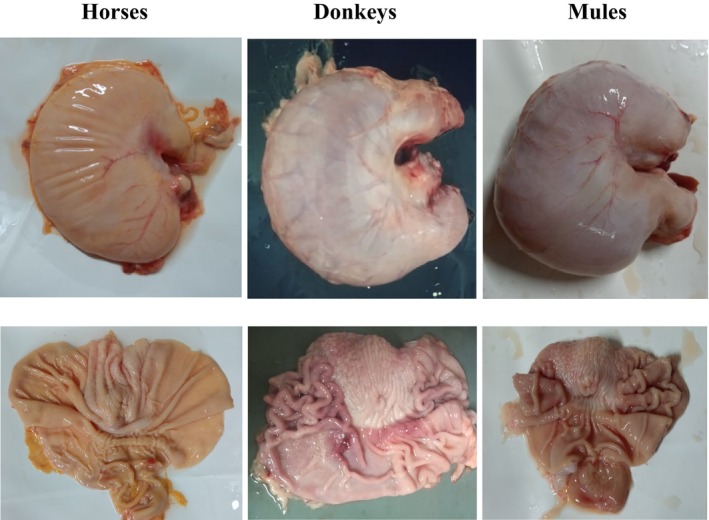
External and internal appearance of stomachs of equid fetuses in the third trimester of gestation last.

Forty‐six stomachs from fetuses, including 21 from horses, 21 from donkeys, and 4 from mules, were evaluated. The distribution by sex, gastric pH, weight, volume, and measurements of stomachs for each equid species are presented in Table 1, and the external and internal appearance of a single individual from each species is shown in Figure 1. All fetuses had gastric contents with pH >5.8, and mules had larger stomachs. Mules had heavier stomachs than horses (*P* = .004), and donkeys had more gastric content than horses (*P* = .03).

**TABLE 1:** Classification by sex and variables evaluated in the 46 stomachs of equids fetuses.

Sex	Fetuses		
	Horses	Donkeys	Mules
Female	12	11	2
Male	9	10	2
*Total*	21	21	4

Variable	Mean (±SD)		
Gastric contents pH	6.6 (±0.8)	7.5 (±0.9)	5.8 (±0.5)
Full stomach weight (g)	106.2 (±29)^a^	142.5 (±69)^ab^	193.3 (±52)^b^
Empty stomach weight (g)	37 (±15)	43.4 (±7)	57 (±11)
Gastric fluid volume (ml)	69.2 (±16)^a^	99.1 (±25)^b^	136.3 (±37)^ab^
Large curvature length (cm)	20.7 (±2)	21.6 (±4)	23 (±3)
Lessen curvature length (cm)	2.2 (±0.5)	2.1 (±1)	2 (±0.2)
Longitudinal axe length (cm)	9.2 (±0.8)	10.1 (±2.7)	10.7 (±1.2)
Transversal axe length (cm)	6.1 (±0.4)	5.8 (±2.1)	6.7 (±0.6)

*Note*: In the same row, means followed by different letters indicate significant differences (*P* < .05).

## 4 MACROSCOPIC FINDINGS

**FIGURE 2:**
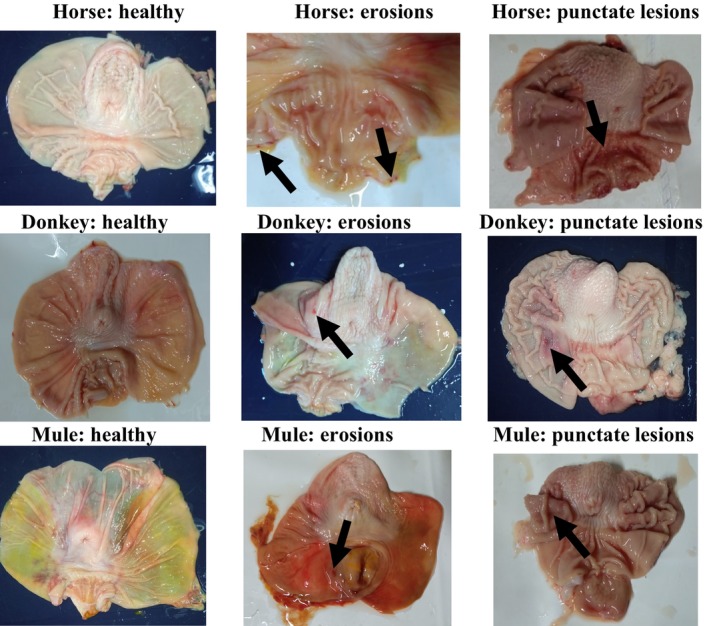
Macroscopic findings in the stomachs of third trimester gestation fetuses.

Macroscopic findings in the evaluated regions of each gastric mucosa are presented in Table 2 and Figure 2. Multifocal erosion was found more commonly in all gastric regions in horse and donkey fetuses than in mule fetuses, followed by areas with hyperemia and punctate lesions observed in all equid species, although not in all evaluated regions. In the cardia region, diffuse hyperemia was less common in horses compared with donkeys (*P* = .05) and mules (*P* = .02). In the pyloric antrum region, diffuse hyperemia was more common in mules than horses (*P* = .02).

**TABLE 2:** Macroscopic findings in the gastric squamous and glandular mucosae of 46 equid creole fetuses.

Region	Horses, n = 21		Donkeys, n = 21		Mules, n = 4	
Squamous	Fundus	Cardia	Fundus	Cardia	Fundus	Cardia
Focal hyperemia and punctate lesions	1	1	2	1	0	0
Multifocal erosion	4	4	2	2	0	0
Diffuse hyperemia	4	0^b^	2	5^a^	2	2^a^

Glandular	Fundus	Pyloric antrum	Fundus	Pyloric antrum	Fundus	Pyloric antrum
Focal hyperemia and punctate lesions	0	5	3	2	1	0
Multifocal erosion	7	4	10	6	0	1
Diffuse hyperemia	6^b^	9	9^ab^	5	4^a^	0

*Note*: In the same row and anatomical site, there is no difference between prevalences followed by the same letter (*P* < .05).

## 5 MICROSCOPIC FINDINGS

**FIGURE 3:**
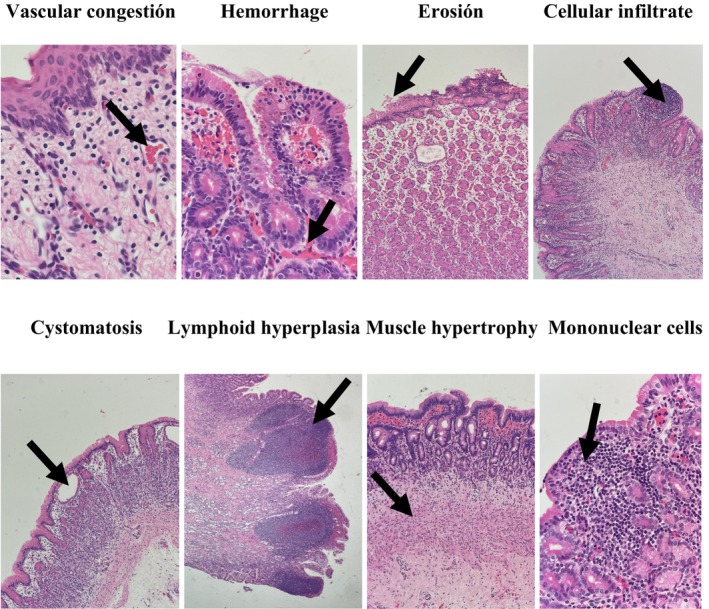
Histopathological lesions found in the stomachs of third‐third gestation fetuses.

Findings from microscopic evaluation of the squamous and glandular gastric mucosa of each fetus are presented in Tables 3 and 4, respectively, and in Figure 3. In the squamous mucosa, all variables were observed with mild degrees in horses. Not all variables were evident in donkeys, but mild, moderate, and severe degrees were found. Meanwhile, mules had some variables in a mild form, with hyperplasia and fibrosis present in fetuses of all 3 equid species, and congestion and mononuclear infiltrates in horses and donkeys. Regarding the glandular mucosa, hyperplasia, congestion, edema, presence of mononuclear cells, exocytosis, fibrosis, and presence of mononuclear cells were observed in fetuses of all 3 equid species in mild and moderate degrees. Mild congestion was more common in donkeys (*P* < .05) than in other species. Meanwhile, hemorrhage and erosion were found in both horses and donkeys, cystomatosis was observed in donkeys and mules, and lymphangiectasia was exclusively noted in horses. In general, higher lesion grades were observed in both mucosae of donkey stomachs but were more pronounced in the glandular mucosa.

**TABLE 3:** Classification of histopathological findings present in the squamous gastric mucosa of 21 Creole equid fetuses.

	Horse, n = 9			Donkey, n = 10			Mule, n = 2		
	Mild	Moderate	Severe	Mild	Moderate	Severe	Mild	Moderate	Severe
*Squamous mucosa*									
Hyperplasia	3	1	—	4	4	1	2	—	—
Congestion	8	—	—	8	1	—	—	—	—
Edema	1	—	—	—	—	—	—	—	—
Polymorphonuclear cells	1	—	—	—	—	—	—	—	—
Mononuclear cells	4	—	—	4	—	—	—	—	—
Fibrosis	2	—	—	7	2	—	2	—	—
*Submucosa*									
Congestion	5	—	—	4	2	—	—	—	—
Fibrosis	1	—	—	2	2	—	2	—	—

**TABLE 4:** Classification of histopathological findings present in the glandular gastric mucosa of 21 Creole equid fetuses.

	Horse, n = 9			Donkey, n = 10			Mule, n = 2		
	Mild	Moderate	Severe	Mild	Moderate	Severe	Mild	Moderate	Severe
Glandular mucosa									
Hyperplasia	4	3	—	2	5	—	1	—	—
Congestion	1	4	—	6*	3	—	2	—	—
Edema	3	—	—	7	—	—	1	—	—
Hemorrhage	2	—	—	4	1	—	—	—	—
Lymphangiectasia	1	—	—	—	—	—	—	—	—
Mononuclear cells	3	1	—	6	2	—	2	—	—
Exocytosis	5	—	—	5	—	—	1	—	—
Erosion	1	—	—	5	—	—	—	—	—
Cystomatosis	—	—	—	1	3	—	—	1	—
Fibrosis	2	—	—	3	—	—	1	—	—
Submucosa									
Congestion	4	—	—	2	4	—	—	—	—
Edema	—	—	—	3	1	—	—	—	—
Mononuclear cells	—	—	—	1	—	—	—	—	—
Fibrosis	1	—	—	1	1	—	1	—	—

* In the same row, differs from equine absolute prevalence for the same grade (*P* < 0.05).

The histopathological diagnoses according to species are listed in Table 5. Based on cellular infiltrate and structural changes in the gastric epithelium, the diagnosis of active chronic congestive gastritis was made in all 3 equid species. Although a significant difference was not detected (*P* = .12), this disease was more common in horse fetuses (6/9), followed by mules (1/2) and donkeys (2/10).

**TABLE 5:** Histopathological diagnoses of the samples from both gastric mucosae of 21 Creole equid fetuses.

	Horses, n = 9	Donkeys, n = 10	Mules, n = 2
Active chronic congestive gastritis	6	2	1
Active chronic hemorrhagic erosive congestive gastritis	1	—	—
Active chronic congestive gastritis with erosion and cystomatosis	—	2	—
Active chronic congestive gastritis with cystomatosis	—	1	1
Active chronic congestive gastritis with erosion	—	2	—
Active chronic congestive follicular gastritis with hemorrhage, erosion, and cystomatosis	—	1	—
Active chronic congestive follicular gastritis	—	1	—
Active chronic follicular gastritis with lymphoid Hyperplasia	—	1	—
Healthy	1	—	—
Insufficient sample	1	—	—

## 6 DISCUSSION

The gestational age of the fetuses allowed for evaluation of all external and intraluminal aspects of the stomach, given that the differentiation of gastric mucosae occurs after the fifth month of gestation.^20^ Most studies on stomach development have focused on the embryonic period (<40 days of gestation), during which the majority of organogenesis occurs.^21^ Therefore, in the final fetal period, morphological changes and allometric growth are less marked, because the stomach must function immediately after birth.^20^ However, our study indicated that equid fetuses in the third trimester of gestation exhibited macroscopic lesions in both gastric mucosae, that were corroborated by histopathology, indicating the need for further study to clarify possible causes or factors involved in the pathophysiology of gastric lesions during fetal development.

Our study did not focus on stomach development in the fetal period, but some results of evaluated variables, despite being within normal ranges, were lower than those reported in a recent study.^20^ This discrepancy may be attributed to the fetuses originating from different breeds of variable weights and sizes. Donkey fetuses, despite donkeys being smaller animals, exhibited a higher degree of liquid distension of their stomachs. Additionally, donkey fetuses had larger stomachs compared with horses, a fact that could be related to intrinsic factors of stomach motility in these animals,^22^, ^23^ as suggested by a previous study reporting a higher number of cases of gastric impaction in adult donkeys among equid species (unpublished data). Furthermore, donkey fetuses had a more alkaline gastric pH than the other species, possibly because of dilutional effects resulting from a higher volume of gastric fluid. However, comparative analysis of the 3 species of equids should be approached with caution, because the low number of mules relative to horses and donkeys may impact statistical power.

The presence of EGUS and its clinical manifestation in adult and geriatric equids have been extensively described in the veterinary literature.^8^, ^11^, ^12^, ^13^, ^14^ It also has been reported in neonates, foals (nursing and weanlings) and young horses, albeit to a lesser extent and with specific clinical presentations for this age group.^15^, ^17^, ^18^, ^24^ However, a previous study did not report the presence of ulcers or other types of gastric lesions in aborted fetuses or those born in dystocic deliveries,^19^ contrasting with our findings. Although ulcers were not evident, macroscopic inspection identified multifocal erosions, diffuse hyperemic areas, and punctate lesions. These findings could be pre‐ulcerative, especially considering that they are prenatal alterations on the gastric surface.

Fetuses in our study came from dams destined for slaughter, subjected to prolonged periods of fasting, extensive travel, and episodes of high stress. This situation arises because Colombia has very few slaughter facilities for equids, and all animals destined for slaughter are subjected to these factors, which have been identified as predisposing factors for EGUS.^9^, ^13^, ^25^, ^26^, ^27^ This population of equid dams showed a high frequency of ESGD and EGGD (unpublished data), compared with the frequency in equids from the same geographical region engaged in other activities.^28^, ^29^ In contrast, previous studies with fetuses^19^ did not describe the status or end‐use of their dams.

The presence of gastric lesions in this fetal population prompts hypotheses to explain their pathophysiology. Because of epitheliochorial placentation, direct contact between maternal blood and the fetal chorion is not possible. This anatomical feature suggests that the transport of exogenous noxious substances from the mother would be unlikely, contrary to what happens with the hemochorial placenta of humans. However, changes in placental and vascular structures have been reported in mares with repercussions on fetal development and growth after systemic diseases and chronic laminitis.^30^, ^31^, ^32^ Similar findings were described in women with systemic diseases and in specific clinical cases.^33^ Therefore, exploring this area is essential to understand possible triggering mechanisms leading to gastric alterations during intrauterine life.^34^

The absence of gestational monitoring, fetal stress assessment, cortisol measurement in both mothers and fetuses, evaluation of uteroplacental attachment, detailed clinical examination, and lack of knowledge of the mothers' previous medical history are limitations of our study and should be explored in the future. The possible roles of endogenous and exogenous factors in the induction of gastric lesions in horse, donkey, and mule fetuses in the last third of gestation also should be studied. However, the contribution of our study is the macroscopic and histopathological description of gastric lesions in fetuses from a specific population the dams of which had gastric ulcers and were subjected to high‐stress conditions.

Several causes of gastric erosion and ulcers in neonates and foals have been described, including physiological stress, hypoxia, alterations in gastric emptying, prolonged fasting, small food portions, long periods of recumbency, systemic diseases, and the use of anti‐inflammatory drugs.^17^, ^35^, ^36^, ^37^, ^38^ However, many of these causes do not apply in intrauterine life, except for physiological stress, and fetal stress was not evaluated in our study. Systemic diseases in the dams were not clinically observed, and there was no evidence of septic foci in fetal membranes during the initial inspection. However, moderate degrees of dehydration in the dams were evident because of prolonged periods of water deprivation, which can alter blood flow and subsequently might affect gastric mucosal defenses.

Although anti‐inflammatory drugs are potential ulcerogenic agents for the gastrointestinal mucosa, primarily through the inhibition of cytoprotective prostaglandins,^39^, ^40^, ^41^ and by inducing oxidative stress,^42^, ^43^ their use could not be evaluated in our study because of the lack of previous medical information on the dams. However, the most affected mucosa, especially in donkey fetuses, was the glandular mucosa, coinciding with EGGD, where anti‐inflammatory drugs are considered inducers.^4^, ^9^ Although anti‐inflammatory drugs also are considered an indirect cause of gastric squamous erosion,^5^ a larger role is attributed to the caustic effect of gastric content with pH <2.^44^, ^45^ However, fetal gastric contents showed an average pH >6, corresponding to mild degrees of lesions found in this area of mucosa. Donkey fetuses with more lesions had more alkaline pH, indicating the possibility that pH may not play a prominent role in the described lesions in the squamous mucosa of these fetuses.

Microscopic findings and histopathological diagnoses of both gastric mucosae in the fetuses are consistent with those described in previous studies of young and adult equids,^29^, ^46^, ^47^, ^48^ with chronic and active inflammatory conditions being the most common findings in the gastric mucosa of equids. However, whether these findings should be considered normal or pathological is unclear, because inflammatory infiltrates are always present in some cases without macroscopic lesions, and few studies correlate macroscopic findings with microscopic findings.^47^ Given that the stomach is constantly exposed to endogenous and exogenous factors after birth, reactions, and activation of defense mechanisms are expected.^20^ Nevertheless, it is surprising to find inflammatory reactions alongside macroscopic findings in fetuses, encouraging future studies.

Ours is the first histopathological report of fetal gastric mucosa in equids, as there is only a single study on squamous mucosa characteristics related to age, with no reports of lesions.^19^ Gastric ulceration in a 24‐hour‐old neonate has been described,^15^, ^18^ and recently, gastritis was described in foals (<5 months) before weaning, which could influence the frequency of gastric lesions in this population.^24^

In conclusion, we have provided evidence of macroscopic and histopathologic lesions in both gastric mucosae of equid fetuses in the last third of gestation. These findings should prompt future studies to determine their pathophysiology, given the characteristics of this population, the necropsy nature of our study and the absence of knowledge of in vivo injury mechanisms. Our results draw attention to areas of perinatology and neonatology regarding the presence and evolution of pre‐ulcerative lesions before birth, which should be considered in the primary care and management of neonates.

## CONFLICT OF INTEREST DECLARATION

Authors declare no conflict of interest.

## OFF‐LABEL ANTIMICROBIAL DECLARATION

Authors declare no off‐label use of antimicrobials.

## INSTITUTIONAL ANIMAL CARE AND USE COMMITTEE (IACUC) OR OTHER APPROVAL DECLARATION

Approved by the Ethics Committee for Experimentation with Animals of the University of Antioquia (No. 1472022), guaranteeing that they were carried out in accordance with the relevant laws and guidelines.

## HUMAN ETHICS APPROVAL DECLARATION

Authors declare human ethics approval was not needed for this study.

## References

[1] Murray MJ , Grodinsky C . Regional gastric pH measurement in horses and foals. Equine Vet J Suppl. 1989;21:73‐76. 10.1111/j.2042-3306.1989.tb05660.x 9118111

[2] Nappert G , Vrins A , Larybyere M . Gastroduodenal ulceration in foals. Compend Contin Educ Pract Vet. 1989;5:11.

[3] Sykes BW , Hewetson M , Hepburn RJ , Luthersson N , Tamzali Y . European College of Equine Internal Medicine Consensus Statement equine gastric ulcer syndrome in adult horses. J Vet Intern Med. 2015;29:1288‐1299. 26340142 10.1111/jvim.13578 PMC4858038

[4] Banse H , Andrews F . Equine glandular gastric disease: prevalence, impact and management strategies. Vet Med. 2019;10:69‐76. 10.2147/VMRR.S174427 PMC6642651 31406687

[5] Hewetson M , Tallon R . Equine squamous gastric disease: prevalence, impact and management. Vet Med. 2021;12:381‐399. 10.2147/VMRR.S235258 PMC8725839 35004264

[6] Begg LM , O'Sullivan CB . The prevalence and distribution of gastric ulceration in 345 racehorses. Aust Vet J. 2003;81:199‐201. 15080440 10.1111/j.1751-0813.2003.tb11469.x

[7] Jonsson H , Egenvall A . Prevalence of gastric ulceration in Swedish Standardbreds in race training. Equine Vet J. 2006;38(3):209‐213. 16706273 10.2746/042516406776866390

[8] McGovern K . Updates on gastric ulceration in adult horses. Livestock. 2017;22(5):272‐277.

[9] Sykes B , Bowen M , Habershon‐Butcher J , Green M , Hallowell GD . Management factors and clinical implications of glandular and squamous gastric disease in horses. J Vet Intern Med. 2019;33:233‐240. 30499188 10.1111/jvim.15350 PMC6335573

[10] Patiño JJ , Vélez SA , Martínez JR . Ethological, endocrinological, and gastroscopic evaluation of crib‐biting Colombian creole horses. J Vet Behav. 2020;40:92‐97.

[11] Vokes J , Lovett A , Sykes B . Equine gastric ulcer syndrome: an update on current knowledge. Animals. 2023;13:1261. 37048517 10.3390/ani13071261 PMC10093336

[12] Van den Boom R . Equine gastric ulcer syndrome in adult horses. Vet J. 2022;283‐284:105830. 10.1016/j.tvjl.2022.105830 35472513

[13] Murray MJ , Schusser GR , Pipers FS , Gross SJ . Factors associated with gastric lesions in thoroughbred racehorses. Equine Vet J. 1996;28(5):368‐374. 8894534 10.1111/j.2042-3306.1996.tb03107.x

[14] Murray MJ . Diseases of the stomach. In: Smith BP , ed. Large Animal Internal Medicine. Vol 3. St. Louis, MO: Mosby Elsevier; 2009:695‐702.

[15] Elfenbein JR , Sanchez LC . Prevalence of gastric and duodenal ulceration in 691 nonsurviving foals (1995‐2006). Equine Vet J Suppl. 2012;41:76‐79. 10.1111/j.2042-3306.2011.00449.x 22594031

[16] Murray MJ , Hart J , Parker GA . Equine gastric ulcer syndrome: endoscopic survey of asymptomatic foals. Proc Am Assoc Equine Pract. 1987;33:769‐776.

[17] Murray M . Pathophysiology of peptic disorders in foals and horses: a review. Equine Vet J. 1999;29:14‐18. 10.1111/j.2042-3306.1999.tb05162.x 10696287

[18] Lewis S . Gastric ulceration in an equine neonate. Can Vet J. 2003;44(5):420‐421. 12757136 PMC340153

[19] Murray MJ , Mahaffey EA . Age‐related characteristics of gastric squamous epithelial mucosa in foals. Equine Vet J. 1993;25(6):514‐517. 8275998 10.1111/j.2042-3306.1993.tb03003.x

[20] Poradowski D , Chrószcz A . Equine stomach development in the fetal period: an anatomical, topographical, and morphometric study. Animals. 2022;12:2966. 36359095 10.3390/ani12212966 PMC9658733

[21] Rodrigues M , Carvalho R , Franciolli A , et al. Prenatal development of the digestive system in the horse. Anat Rec. 2014;297:1218‐1227. 10.1002/ar.22929 24778084

[22] Freeman DE . Gastric impaction. Equine Vet Educ. 2011;23:174‐176.

[23] Blikslager AT . Gastric impaction and large colon volvulus: can one lead to the other? Equine Vet Educ. 2015;27:460‐461.

[24] Campos de Araújo ÂM , Da Silva AH , Bastos FL , Seidner JT , et al. Influence of weaning management on gastritis incidence in foals. J Equine Vet. 2022;113:103917. doi:10.1016/j.jevs.2022.103917 35218905

[25] Luthersson N , Nielsen KH , Harris P , Parkin TD . Risk factors associated with equine gastric ulceration syndrome (EGUS) in 201 horses in Denmark. Equine Vet J. 2009b;41(7):625‐630. 19927579 10.2746/042516409x441929

[26] Pedersen SK , Cribb AE , Windeyer MC , Read EK , French D , Banse HE . Risk factors for equine glandular and squamous gastric disease in show jumping Warmbloods. Equine Vet J. 2018;50(6):747‐751. 29660168 10.1111/evj.12949

[27] Padalino B , Davis GL , Raidal SL . Effects of transportation on gastric pH and gastric ulceration in mares. J Vet Intern Med. 2020;34:922‐932. 32009244 10.1111/jvim.15698 PMC7096603

[28] Zuluaga AM , Ramírez NF , Martínez JR . Equine gastric ulcerative syndrome in Antioquia (Colombia): frequency and risk factors. Rev Colomb Cienc Pecu. 2018;31(2):139‐149.

[29] Calixto LC , Martínez JR . Gastroscopic characterization and prevalence of gastric ulcer syndrome in working mules in Colombia. Equine Vet J. 2023;56:449‐455. doi:10.1111/evj.13985 37559426

[30] Rossdale PD . The maladjusted foal: influences of intrauterine growth retardation and birth trauma. Proc Am Assoc Equine Pract. 2004;50:75‐126.

[31] Wilsher S , Allen WR . Effects of a streptococcus equi infection mediated nutritional insult during mid‐gestation in thoroughbred mares. I Placental and fetal development. Equine Vet J. 2006;38:549‐557. 17124846 10.2746/042516406x156497

[32] Pazinato F , da Rosa B , Fernandes C , et al. Histomorphometry of the placental vasculature and microcotyledons in thoroughbred mares with chronic laminitis. Theriogenology. 2017;91:77‐81. 28215689 10.1016/j.theriogenology.2016.12.009

[33] Mayhew TM . Patterns of villous and intervillous space growth in human placentas from normal and abnormal pregnancies. Eur J Obstet Gynecol Reprod Biol. 1996;68(1‐2):75‐82. 8886685 10.1016/0301-2115(96)02486-4

[34] Rossdale PD , Ousey JC , Chavatte P . Readiness for birth: an endocrinological duct between fetal foal and mare. Equine Vet J Suppl. 1997;24:96‐99. 10.1111/j.2042-3306.1997.tb05085.x 9355809

[35] Sanchez LC , Lester GD , Merritt AM . Effect of ranitidine on intragastric pH in clinically normal neonatal foals. J Am Vet Med Assoc. 1998;212:1407‐1412. 9589127

[36] Breuhaus BA , DeGraves FJ , Honore EK , Papich MG . Pharmacokinetics of ibuprofen after intravenous and oral administration and assessment of safety of administration to healthy foals. Am J Vet Res. 1999;60:1066‐1073. 10490073

[37] Merritt AM . Normal equine gastroduodenal secretion and motility. Equine Vet J. 1999;31(S29):7‐13. 10.1111/j.2042-3306.1999.tb05161.x 10696286

[38] Murray MJ . Gastroduodenal ulceration in foals. Equine Vet Educ. 1999;11:199‐207.

[39] MacAllister C , Morgan S , Borne A , Pollet R . Comparison of adverse effects of phenylbutazone, flunixin meglumine and ketoprofen in horse. J Am Vet Assoc. 1993;202:71‐77. 8420909

[40] Lanas A , Panés J , Pique JM . Clinical implications of COX‐1 and/or COX‐2 inhibition for the distal gastrointestinal tract. Curr Pharm des. 2003;9(27):2253‐2266. 14529405 10.2174/1381612033453992

[41] Tomlinson J , Blikslager A . Role of nonsteroidal anti‐inflammatory drugs in gastrointestinal tract injury and repair. J Am Vet Med Assoc. 2003;222(7):946‐951. 12685784 10.2460/javma.2003.222.946

[42] Naito Y , Yoshikawa T , Yoshida N , Kondo M . Role of oxygen radical and lipid peroxidation in indomethacin‐induced gastric mucosal injury. Dig Dis Sci. 1998;43(9 Suppl):30S‐34S. 9753223

[43] Martínez JR , Cândido de Andrade BS , Silveira Alves GE . Orally administered phenylbutazone causes oxidative stress in the equine gastric mucosa. J Vet Pharmacol Ther. 2014;38(3):257‐264. 25287371 10.1111/jvp.12168

[44] Lorenzo M , Merritt AM . Effects of exercise on gastric volume and pH in the proximal portion of the stomach of horses. Am J Vet Res. 2002;63(11):1481‐1487. 12428654 10.2460/ajvr.2002.63.1481

[45] Husted L , Sanchez LC , Baptiste KE , et al. Effect of a feed/fast protocol on pH in the proximal equine stomach. Equine Vet J. 2009;41:658‐662. 19927584 10.2746/042516409x416431

[46] Martineau H , Thompson H , Taylor D . Pathology of gastritis and gastric ulceration in the horse. Part 1: range of lesions present in 21 mature individuals. Equine Vet J. 2009a;41(7):638‐644. 19927581 10.2746/042516409x464816

[47] Martineau H , Thompson H , Taylor D . Pathology of gastritis and gastric ulceration in the horse. Part 2: a scoring system. Equine Vet J. 2009;41(7):646‐651. 19927582 10.2746/042516409x464799

[48] Al‐Mokaddem AK , Ahmed KA , Doghaim RE . Pathology of gastric lesions in donkeys: a preliminary study. Equine Vet J. 2014;47:1‐5. 10.1111/evj.12336 25138464

